# Supplementary material for: Transcriptomic Analysis Reveals the High-Oleic Acid Feedback Regulating the Homologous Gene Expression of Stearoyl-ACP Desaturase 2 (SAD2) in Peanuts
Source: Int J Mol Sci. 2019 Jun 25;20(12):3091. doi: 10.3390/ijms20123091 (PMC6628111; doi:10.3390/ijms20123091)
Supplement: Supplementary file 1 [file ijms-20-03091-s001.zip › SP files/SP file transcript OA.pdf]

Title:

Transcriptomic Analysis Reveals the High-oleic Acid Feedback Regulating the Homologous Gene Expression of Stearoyl-ACP Desaturase 2 (*SAD2*) in Peanut

Author names:

Hao Liu[1#], Jianzhong Gu[2#], Qing Lu[1], Haifen Li[1], Yanbin Hong [1], Xiaoping Chen[1], Li Ren[2], Li Deng[2], Xuanqiang Liang[1*]

# For co-first author, these authors contributed equally to this work.

* For corresponding.

Affiliations:

[1] Crops Research Institute, Guangdong Academy of Agricultural Sciences, Guangdong Provincial Key Laboratory of Crop Genetic Improvement, South China Peanut Sub-Center of National Center of Oilseed Crops Improvement, Guangzhou, 510640, China.

[2] Peanut Research Institute, Kaifeng Academy of Agricultural Sciences, Kaifeng 475004, China.

***Corresponding author:**

Xuanqiang Liang,

E-mail: liangxuanqiang@gdaas.cn

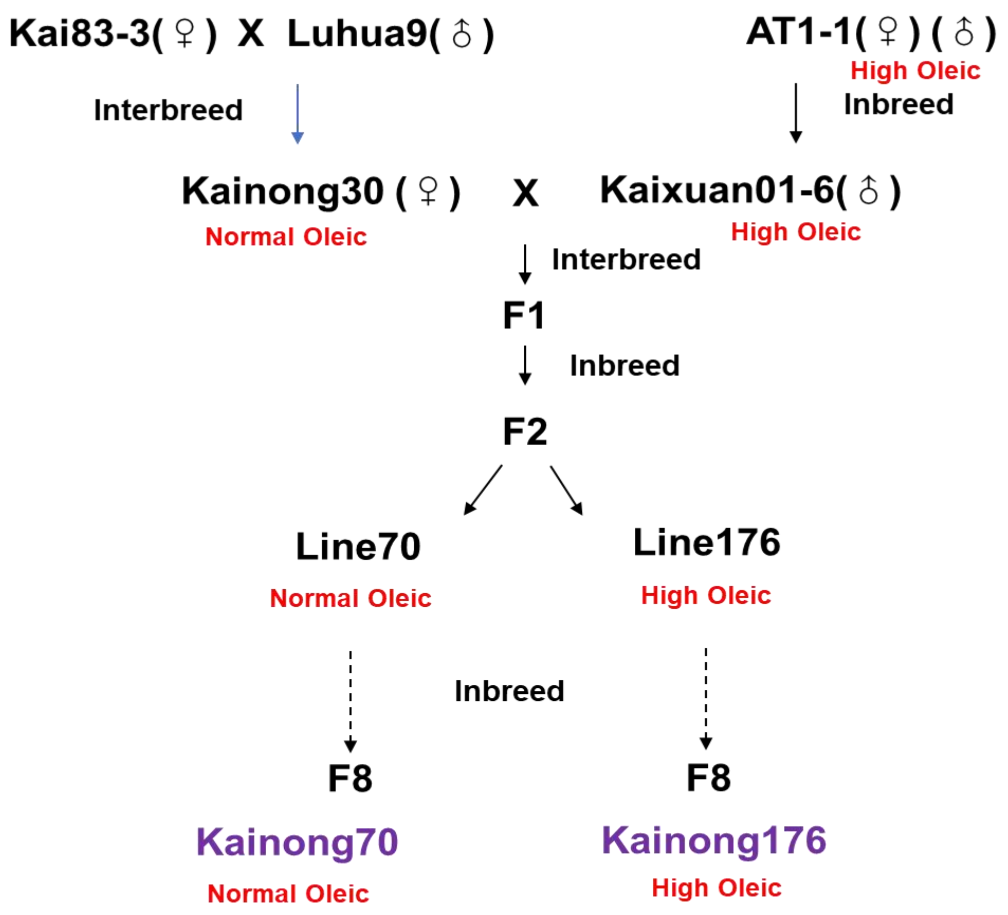

Figure S1. The breeding processes of normal- and high-oleic cultivars Kainong70 and Kainong176. Kai83-3(♀) intercrossed with Luhua9(♂) to cultivate the kainong30, Kaixuan01-6 selected from the inbreeding population of American introduced high-oleic peanut variety AT1-1(♀) (♂). Kainong30 (♀) intercrossed with Kaixuan01-6 (♂) to generate the F2 population, normal- and high-oleic line70 and 176 isolated from the F2 population, and self-fertilized to generate the normal-oleic cultivar Kainong70 and high-oleic cultivar Kainong176 until the stable F8 population. These varieties information provided by the website of www.peanutdata.cn.

>*FAD2-A* coding sequence in high-oleate cultivar kainong176

ATGGGAGCTGGAGGGCGTGTCACTAAGATTGAAGCTCAAAAGAAGCCTCTTTCAAGG
GTTCCACATTCAAACCCTCCATTCAGTGTTGGCCAACTCAAGAAAGCAATTCCACCAC
ATTGCTTTGAACGTTCTCTTTTCATATCATTCTCCTATGTTGTCTATGATCTCTTAGTGGC
CTACTTACTCTTCTACATTGCCACCACTTATTTCCACAAGCTTCCATACCCATTTTCCTTC
CTTGCTTGGCCAATCTATTGGGCCATCCAAGGCTGCATTCTCACTGGTGTTTGGGTGAT
TGCTCATGAGTGTGGCCACCATGCCTTCAGCAAGTACCAACTTGTTGATGACATGGTTG
GTTTGACCCTTCACTCTTGA(375bp)CTATTAGTTCCTTATTTCTCATGGAAAATCAGCCA
CCGCCGCCACCACTCCAACACCGGTTCCCTCGACCGCAACGAAGTGTTTGTCCCAAAA
CCAAAATCAAAGGTATCATGGTATAACAAGTACATGAACAATCCACCAGGGAGGGCTAT
CTCCCTCTTCATCACACTCACACTAGGATGGCCCTTGTACTTGGCCTTCAATGTTTCTGG
CAGACCCTATGATAGATTTGCAAGCCACTATGACCCTTATGCTCCCATATACTCTAACAG
GGAAAGGCTTCTAATTTATGTCTCAGATTCATCTGTCTTTGCTGTAACATATCTGCTATAT
CACATAGCAACTTTGAAAGGTTTGGGTTGGGTGGTATGTGTTTATGGGGTGCCATTGCT
CATTGTGAATGGGTTTCTAGTTACCATAACCTATTTGCAGCACACACATGCATCATTGAC
TCACTATGATTCATCCGAATGGGACTGGTTAAGAGGAGCATTGGCAACAGTGGACAGA
GATTATGGGATACTGAATAAGGCATTTCATCATATAACTGATACGCATGTGGCTCATCATT
TGTTCTCAACAATGCCTCATTACCATGCAATGGAAGCAACCAATGCAATAAAGCCAATA
TTGGGTGATTACTACCAATTTGATGGCACCCCAGTTTACAAAGCATTGTGGAGAGAAGC
CAAAGAGTGCCTCTATGTGGAGCCAGATGATGGAGCTTCTCAGAAGGGTGTTTATTGGT
ACAAGAACAAGTTCTGA

>*FAD2-A* in normal-oleate cultivar kainong70

ATGGGAGCTGGAGGGCGTGTCACTAAGATTGAAGCTCAAAAGAAGCCTCTTTCAAGG
GTTCCACATTCAAACCCTCCATTCAGTGTTGGCCAACTCAAGAAAGCAATTCCACCAC
ATTGCTTTGAACGTTCTCTTTTCATATCATTCTCCTATGTTGTCTATGATCTCTTAGTGGC
CTACTTACTCTTCTACATTGCCACCACTTATTTCCACAAGCTTCCATACCCATTTTCCTTC
CTTGCTTGGCCAATCTATTGGGCCATCCAAGGCTGCATTCTCACTGGTGTTTGGGTGAT
TGCTCATGAGTGTGGCCACCATGCCTTCAGCAAGTACCAACTTGTTGATGACATGGTTG
GTTTGACCCTTCACTCTTGTCTATTAGTTCCTTATTTCTCATGGAAAATCAGCCACCGCC
GCCACCACTCCAACACCGGTTCCCTCGACCGCGACGAAGTGTTTGTCCCAAAACCAA
AATCAAAGGTATCATGGTATAACAAGTACATGAACAATCCACCAGGGAGGGCTATCTCC
CTCTTCATCACACTCACACTAGGATGGCCCTTGTACTTGGCCTTCAATGTTTCTGGCAG
ACCCTATGATAGATTTGCAAGCCACTATGACCCTTATGCTCCCATATACTCTAACAGGGA
AAGGCTTCTAATTTATGTCTCAGATTCATCTGTCTTTGCTGTAACATATCTGCTATATCAC
ATAGCAACTCTGAAAGGTTTGGGTTGGGTGGTATGTGTTTATGGGGTGCCATTGCTCAT
TGTGAATGGGTTTCTAGTTACCATAACCTATTTGCAGCACACACATGCATCATTGCCTCA
CTATGATTCATCCGAATGGGACTGGTTAAGAGGAGCATTGGCAACAGTGGACAGAGAT
TATGGGATACTGAATAAGGCATTTCATCATATAACTGATACGCATGTGGCTCATCATTTGT
TCTCAACAATGCCTCATTACCATGCAATGGAAGCAACCAATGCAATAAAGCCAATATTG
GGTGATTACTACCAATTTGATGGCACCCCAGTTTACAAAGCATTGTGGAGAGAAGCCA
AAGAGTGCCTCTATGTGGAGCCAGATGATGGAGCTTCTCAGAAGGGTGTTTATTGGTAC
AAGAACAAGTTCTGA

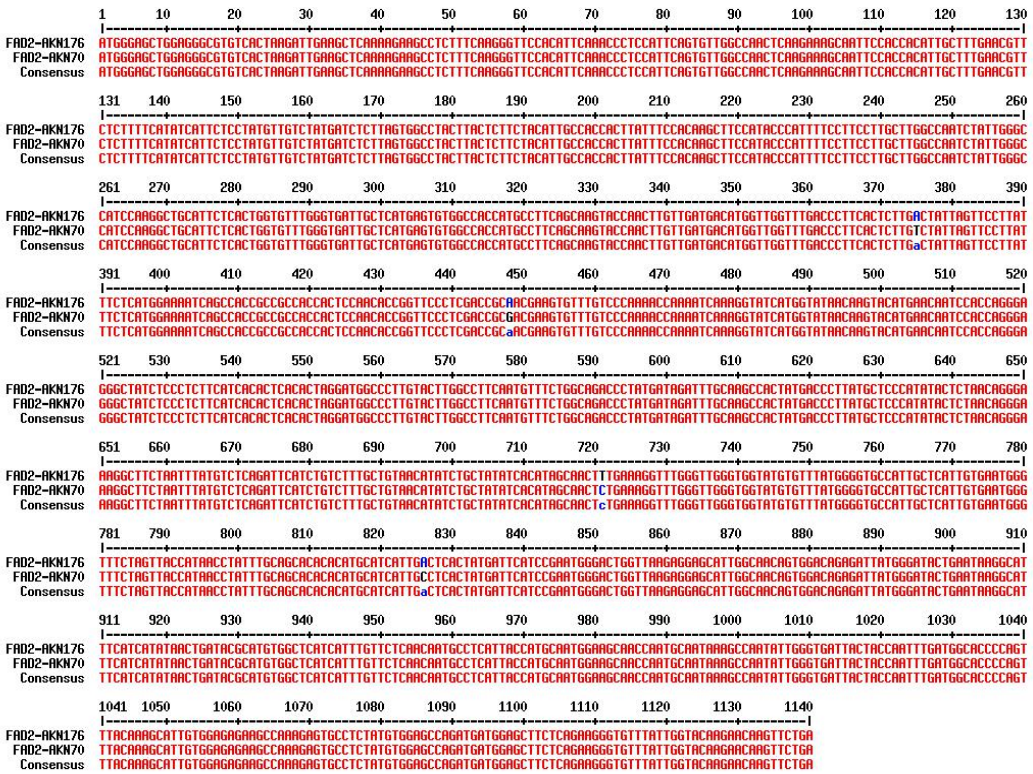

>*FAD2-B* coding sequence in kainong176

ATGGGAGCTGGAGGGCGTGTCACTAAGATTGAAGCTCAAAAGAAGCCTCCTTCAAGG
GTTCCACATTCAAACCCTCCATTCAGTGTTGGCCAACTCAAGAAGGCAATTCCACCAC
ATTGCTTTGAACGTTCTCTTTTCATATCATTCTCATATGTTGTCTATGATCTCTTAATGGCC
TACTTACTCTTCTACATTGCCACCACTTATTTCCACAAGCTTCCATACTCATTATCCTTCC
TTGCTTGGCCAATCTATTGGGCCATCCAAGGCTGCATTCTCACCGGTGTTTGGGTGATT
GCTCATGAGTGTGGCCACCATGCCTTCAGCAAGTACCAACTTGTTGATGACATGGTTGG
TTTGACCCTTCACTCTTGTCTATTAGTTCCTTATTTCTCGTGGAAAATCAGCCACCGCCG
CCACCACTCCAACACAGGTTCCCTCA(442bp)GACCGCGACGAAGTGTTTGTCCCGAAA
CCAAAATCAAAGGTATCATGGTATAACAAGTACATGAACAATCCACCAGGGAGGGCTAT
TTCCCTTTTCATCACACCCACACTAGGATGGCCCTTGTACTTGGCCTTCAATGTTTCTGG
CAGACCCTATGATAGATTTGCAAGCCACTATGACCCTTATGCTCCCATATACTCTAACAG
GGAAAGGCTTCTAATTTATGTCTCAGATTCATCTGTCTTTGCTGTAACATATCTGCTATAT
CACATAGCAACTTTGAAAGGTTTGGGTTGGGTGGTATGTGTTTATGGGGTGCCATTGCT
CATTGTGAATGGGTTTCTAGTTACCATAACCTATTTGCAGCACACACATGCAGCATTGCC
TCACTATGATTCATCCGAATGGGACTGGTTAAGAGGAGCATTGGCAACAGTGGACAGA
GATTATGGGATACTGAATAAGGCATTTCATCATATAACTGATACGCATGTGGCTCATCATT
TGTTCTCAACAATGCCTCATTACCATGCAATGGAAGCAACCAATGCAATAAAGCCAATA
TTGGGTGATTACTACCAATTTGATGGCACCCCAGTTTACAAAGCATTGTGGAGAGAAGC
CAAAGAGTGCCTCTATGTGGAGCCAGATGATGGAGCTTCTCAGAAGGGTGTTTATTGGT
ACAAGAACAAGTTCTGA

>*FAD2-B* coding sequence in kainong70

ATGGGAGCTGGAGGGCGTGTCACTAAGATTGAAGCTCAAAAGAAGCCTCTTTCAAGG
GTTCCACATTCAAACCCTCCATTCAGTGTTGGCCAACTCAAGAAAGCAATTCCACCAC

ATTGCTTTGAACGTTCTCTTTTCATATCATTCTCATATGTTGTCTATGATCTCTTAATGGCC
TACTTACTCTTCTACATTGCCACCACTTATTTCCACAAGCTTCCATACCCATTTTCCTTCC
TTGCTTGGCCAATCTATTGGGCCATCCAAGGCTGCATTCTCACCGGTGTTTGGGTGATT
GCTCATGAGTGTGGCCACCATGCCTTCAGCAAGTACCAACTTGTTGATGACATGGTTGG
TTTGACCCTTCACTCTTGTCTATTAGTTCCTTATTTCTCATGGAAAATCAGCCACCGCCG
CCACCACTCCAACACAGGTTCCCTCGACCGCGACGAAGTGTTTGTCCCGAAACCAAA
ATCAAAGGTATCATGGTATAACAAGTACATGAACAATCCACCAGGGAGGGCTATTTCTC
TTTTCATCACACTCACACTAGGATGGCCCTTGTACCTGGCCTTCAATGTTTCTGGCAGA
CCCTATGATAGATTTGCAAGCCACTATGACCCTTATGCTCCCATATACTCTAACAGGGAA
AGGCTTCTAATTTATGTCTCAGATTCATCTGTCTTTGCTGTAACATATCTGCTATATCACA
TAGCAACTTTGAAAGGTTTGGGTTGGGTGGTATGTGTTTATGGGGTGCCATTGCTCATT
GTGAATGGGTTTCTAGTTACCATAACCTATTTGCAGCACACACATGCATCATTGCCTCAC
TATGATTCATCCGAATGGGACTGGTTAAGAGGAGCATTGGCAACAGTGGACAGAGATT
ATGGGATACTGAATAAGGCATTTCATCATATAACTGATACGCATGTGGCTCATCATTTGTT
CTCAACGATGCCTCATTACCATGCAATGGAAGCAACCAATGCAATAAAGCCAATATTGG
GTGATTACTACCAATTTGATGGCACCCCAGTTTACAAAGCATTGTGGAGAGAAGCCAA
AGAGTGCCTCTATGTGGAGCCAGATGATGGAGCTTCTCAGAAGGGTGTTTATTGGTACA
AGAACAAGTTCTGA

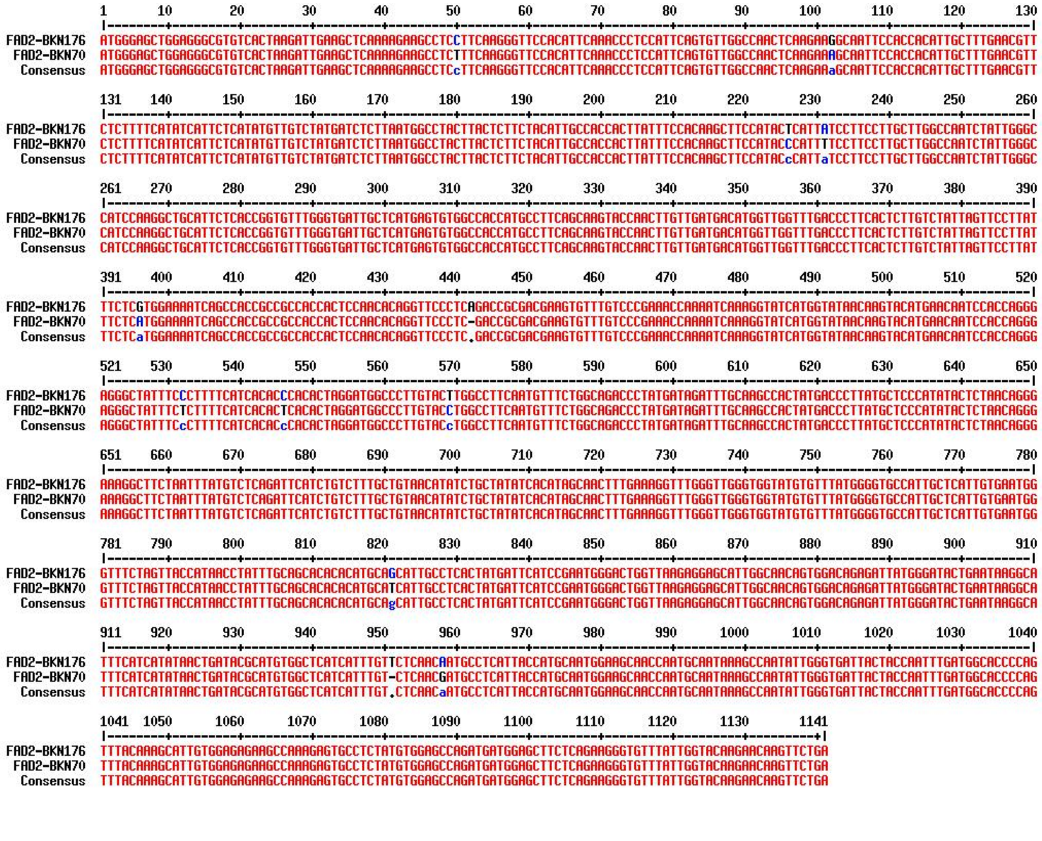

Figure S2. Coding sequence of *FAD2-A* and *FAD2-B* in kainong176 and kainong70.

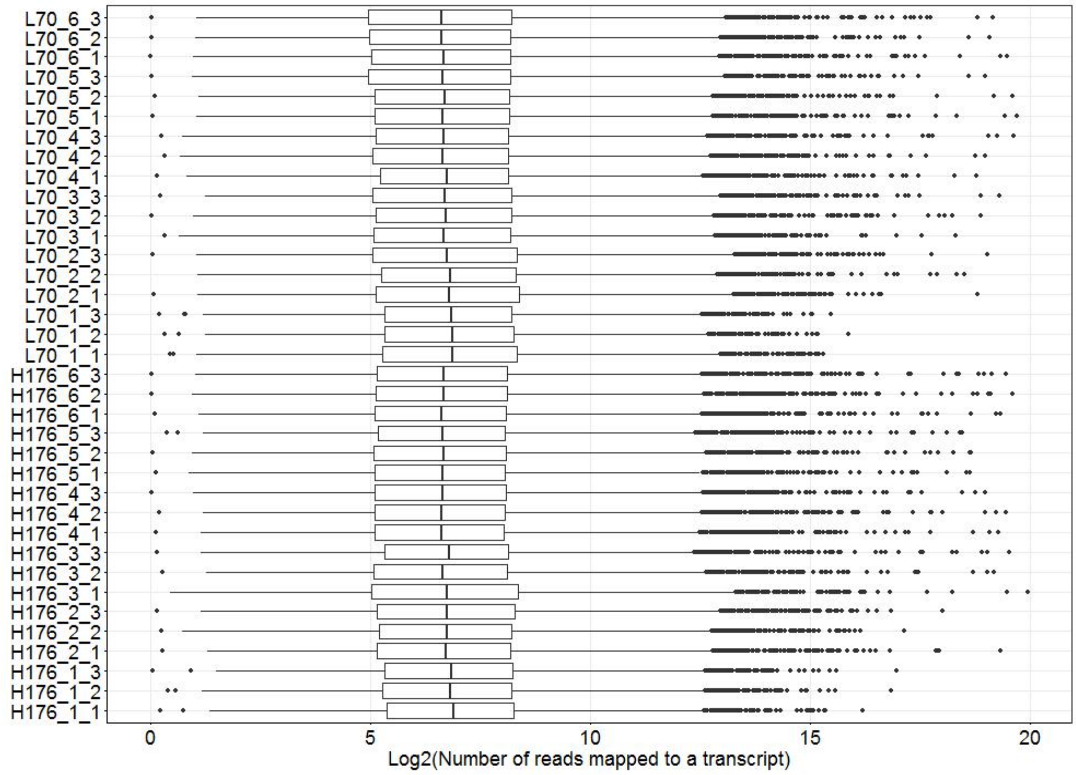

Figure S3. Statistical analysis of assembly reads mapped to the transcript.

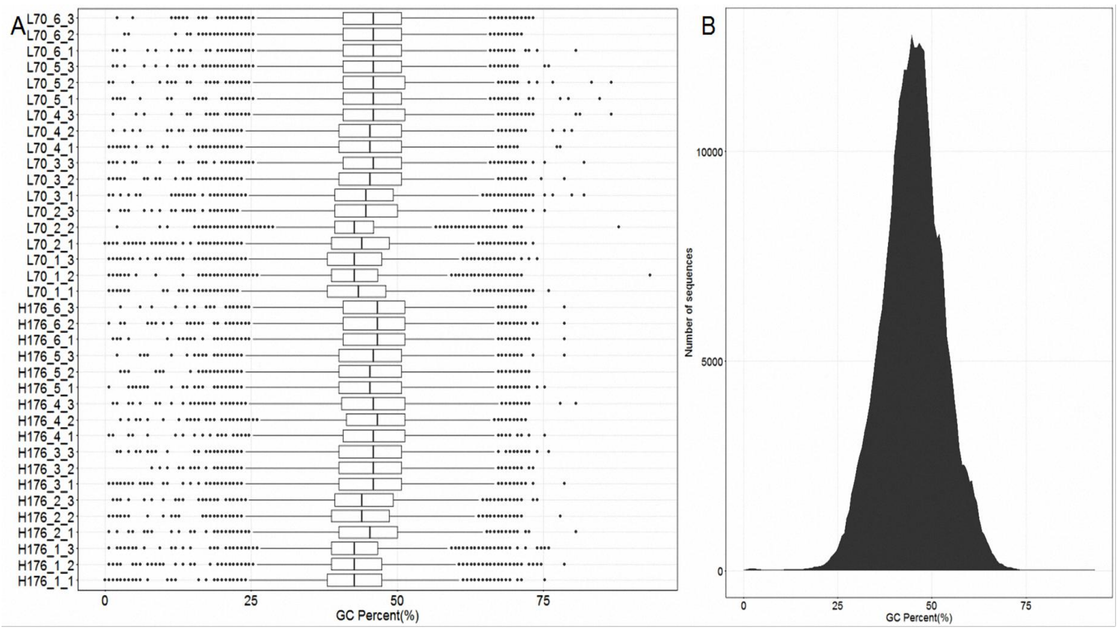

Figure S4. GC content analysis of peanut transcripts. A, Frequency of GC content of peanut transcripts. B, Distribution of GC content of transcripts for peanut transcriptome-seq.

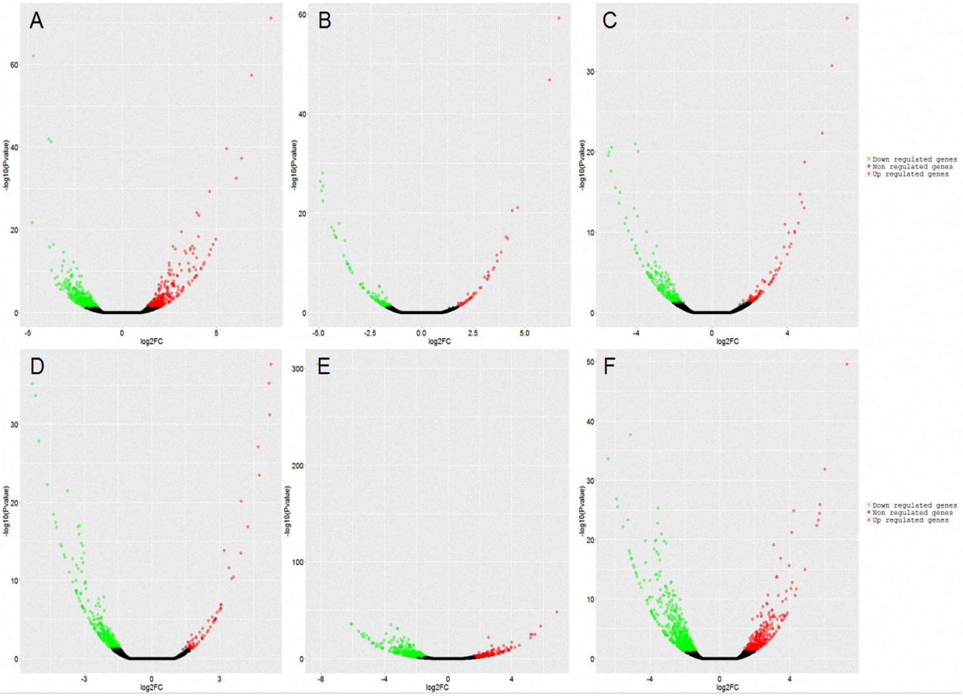

Figure S5. Volcano plot displayed the screening criteria of total DEGs at different seed development stages. A-F represented from 20DAF to 70DAF, respectively.

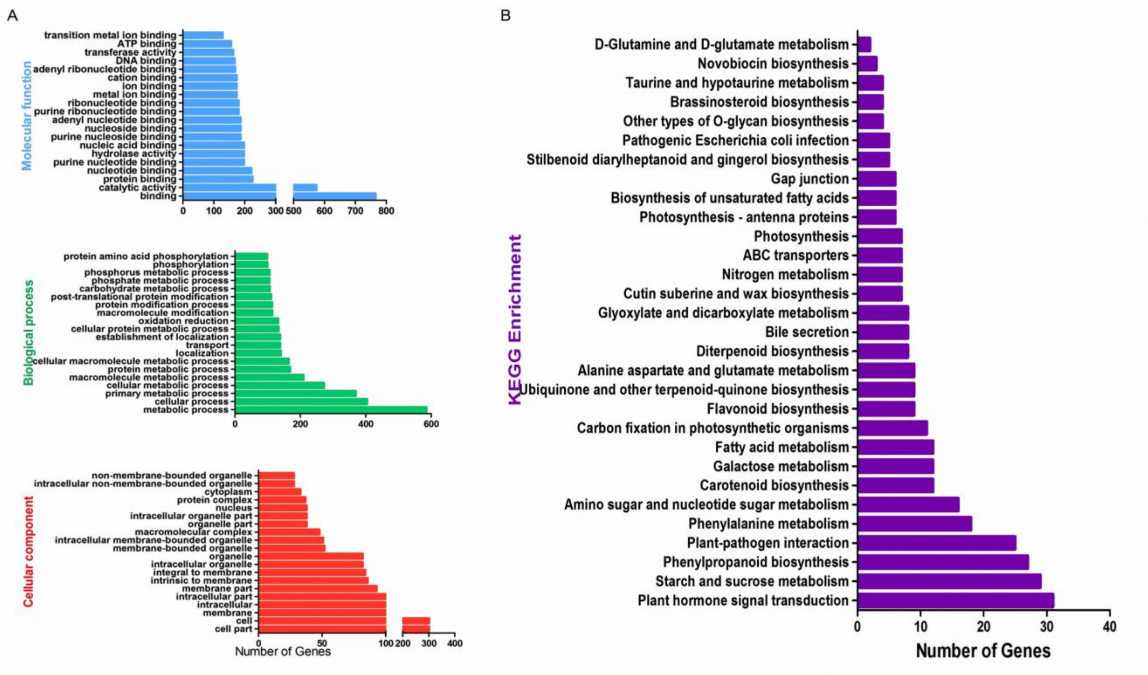

Figure S6. Bioinformatics and statistical analyses of differentially expressed genes. A, GO analysis of the total DEGs in molecular function, biological process, and cellular component, respectively (Top 20 terms). B, KEGG pathway enrichment analysis of DEGs at different seed developmental stages in H176 vs. L70 (Top 30 terms).

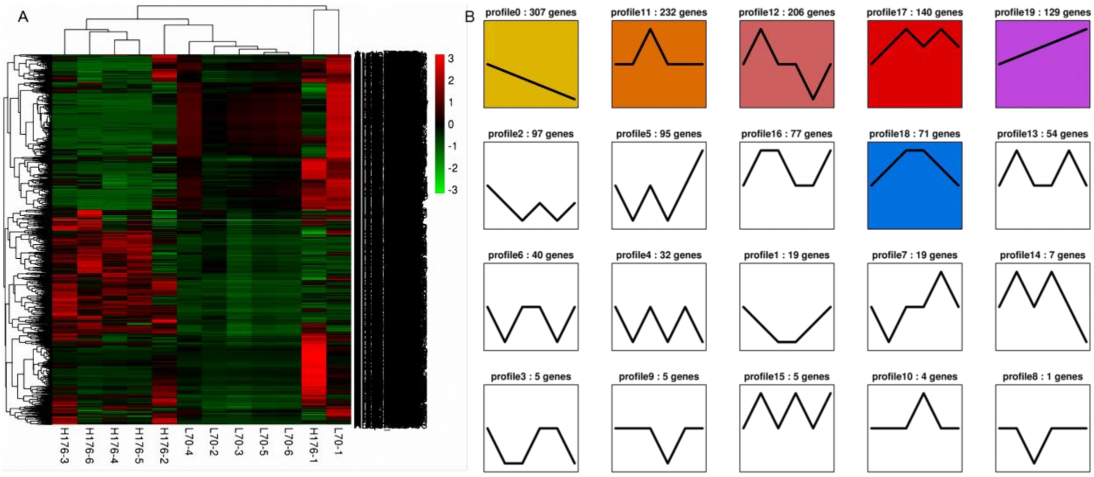

Figure S7. Expression analysis of DEGs between six developmental stages. A, Heatmap showing the relative expression of total DEGs at each stage in L70 and H176. B, Trend analysis of DEGs expression (20 trends). Colored block trend: significant enrichment trend (P<0.05). Without color trend: the enrichment of significant trends.

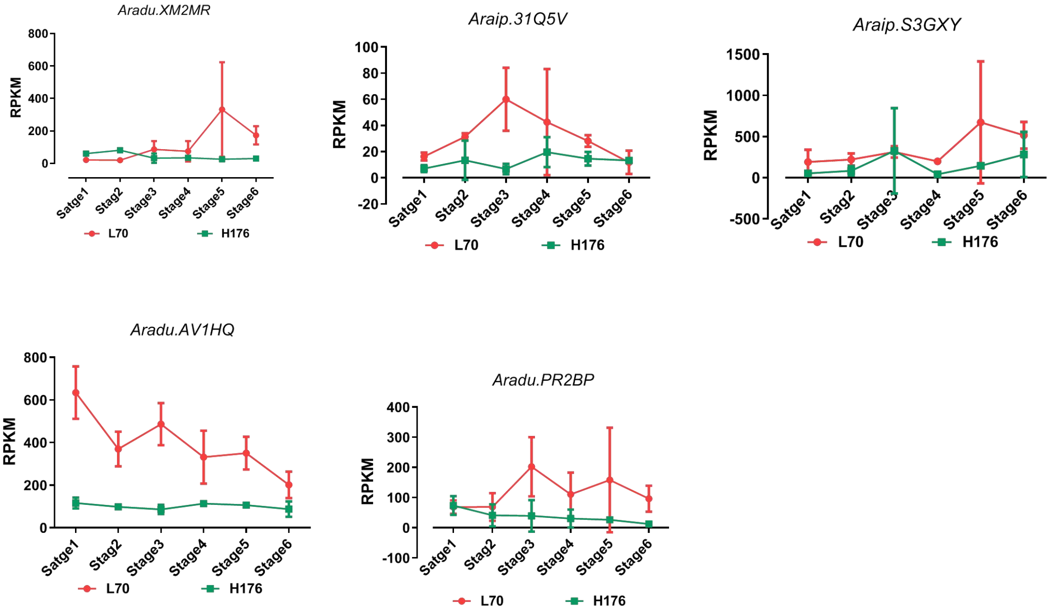

Figure S8. Relative expression levels of lipid DEGs measured by RNAseq.

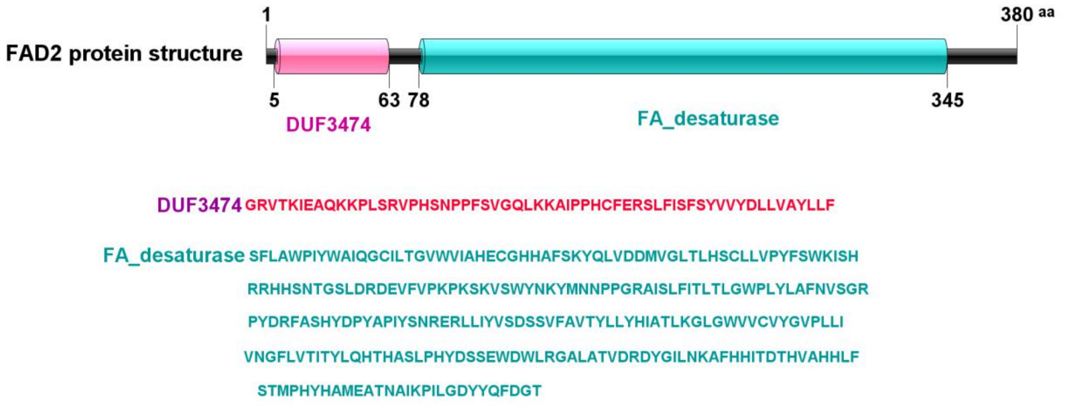

Figure S9. FAD2 protein structure in peanut. FAD2 protein structure predicted by the online database SMART (http://smart.embl-heidelberg.de). The FAD2 protein contains two major conserved domains DUF3474 (5-63aa) and FA-desaturase (78-345aa). DUF3474 indicated the domain of unknown function 3474, this domain is found in fatty acid desaturases (FADs), mainly from plants and fungi. FA indicated the fatty acid, and aa indicated the amino acid.

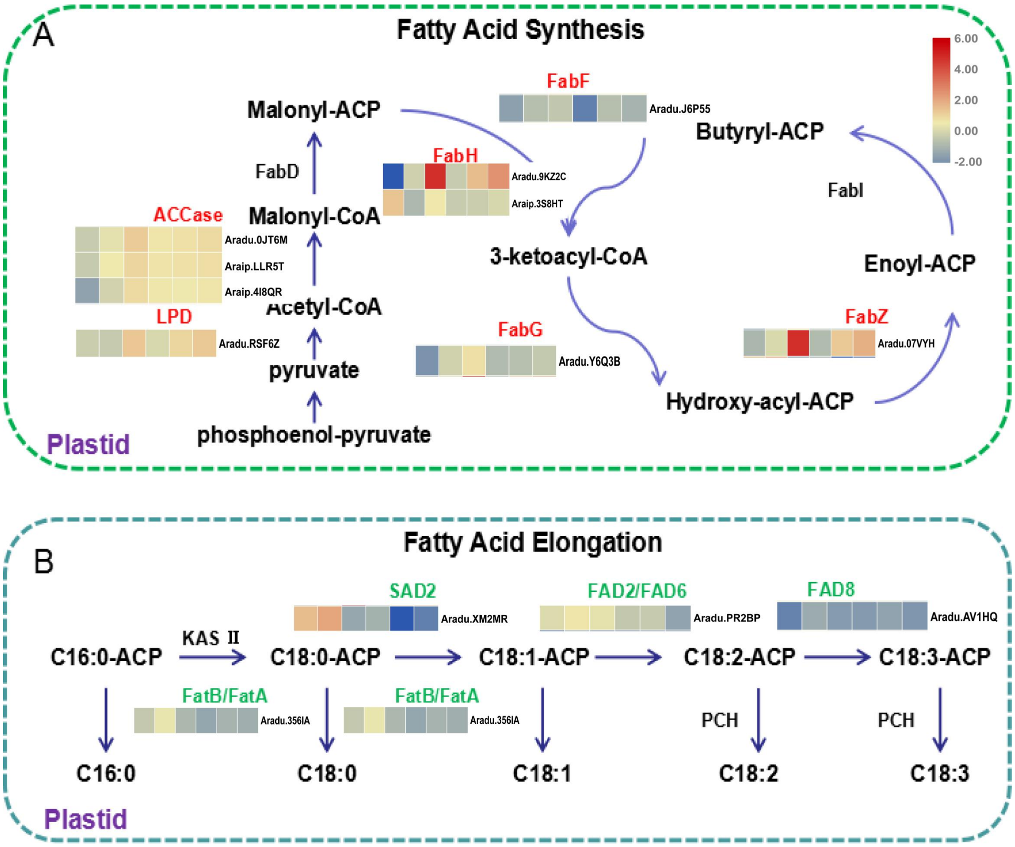

Figure S10. The contrapuntal patterns of lipid DEGs in developing high-oleic peanut seeds. A, identified lipid DEGs are annotated into fatty acid synthesis pathway, the icon is consisted of six blocks that represented six time points of seed development, the icons under the enzyme name show the number of RPKM corresponding to the relative expression of enzyme during high-oleic peanut seed development when compared with L70. B, lipid DEGs are annotated into fatty acid elongation pathway, the icons under the enzyme name show the number of RPKM corresponding to the relative expression of enzyme in H176. Enzyme name with green color indicates the down-regulated gene, and enzyme name with red color represents the up-regulated gene.

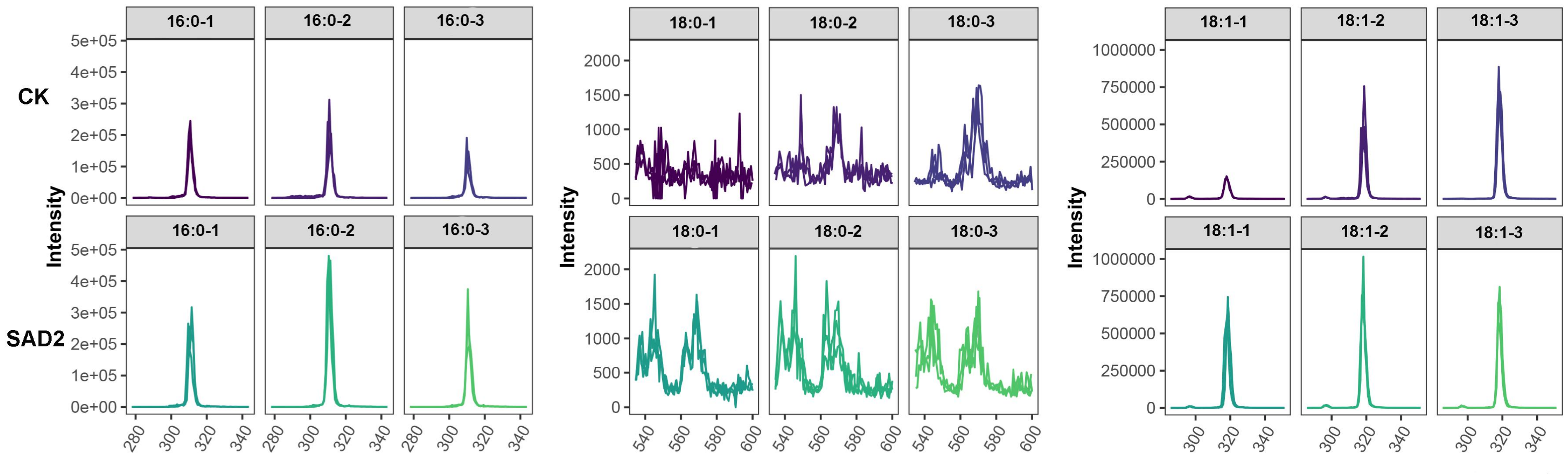

Figure S11. Mass spectrum of C16:0, C18:0, and C18:1 in ectopic expressed yeast cells of *SAD2* (*Aradu.XM2MR*), respectively.
